# Neural Drive Impairment in Chronic Kidney Disease Patients Is Associated with Neuromuscular Fatigability and Fatigue

**DOI:** 10.1249/MSS.0000000000003090

**Published:** 2022-12-13

**Authors:** ANTOINE CHATRENET, GIORGINA PICCOLI, AGATHE ANTHIERENS, MASSIMO TORREGGIANI, JEAN MICHEL AUDEBRAND, BAPTISTE MOREL, BRUNO BEAUNE, SYLVAIN DURAND

**Affiliations:** 1 Le Mans Université, Movement–Interactions–Performance, Le Mans, FRANCE; 2 Nephrology, Centre Hospitalier Le Mans, Le Mans, FRANCE; 3 Endocrinology, Centre Hospitalier Le Mans, Le Mans, FRANCE; 4 Inter-University Laboratory of Human Movement Biology, Université Savoie Mont Blanc, Chambéry, FRANCE

**Keywords:** MUSCLE FATIGUE, PREDIALYSIS, TIREDNESS, PATIENT REPORTED OUTCOMES, RATE OF EMG RISE

## Abstract

**Introduction:**

Chronic kidney disease (CKD) patients have a high degree of fatigue relating to neuromuscular symptoms. There is a lack of evidence regarding the etiology of neuromuscular fatigability in elderly CKD patients.

**Methods:**

Inclusion criteria are as follows: age ≥60 yr, glomerular filtration rate (GFR) <45 mL·min^−1^ per 1.73 m^2^ in CKD patients, and GFR >60 mL·min^−1^·1.73 m^−2^ in controls. The fatigability protocol consisted in a submaximal handgrip task at 40% peak force. Fatigue was assessed using the Multidimensional Fatigue Inventory–20 items (MFI-20) and the Functional Assessment of Chronic Illness Therapy–Fatigue questionnaires. Peak rate of force development (RFD_peak_, normalized: NRFD_peak_) and rate of EMG rise (RER) were measured during explosive contractions; peak force and mean surface EMG were measured during maximum voluntary contractions. Multilevel models tested neuromuscular parameters adjusted for clinical and Multidimensional Fatigue Inventory–20 items subscales. Neuromuscular fatigability contribution to fatigue description was tested using model comparison.

**Results:**

The study included 102 participants; 45 CKD patients and 57 controls. CKD mainly affected the mental and the reduced motivation subscales of fatigue. CKD was associated with greater neuromuscular fatigability assessed using NRFD_peak_ (group–time interaction, −16.7 % MVF·s^−1^, *P* = 0.024), which increased with fatigue severity (*P* = 0.018) and with a higher rate of decrement in RER compared with controls (RER at 50 ms: *β* = −121.2 μV·s^−1^, *P* = 0.016, and *β* = −48.5 μV·s^−1^, *P* = 0.196, respectively). Furthermore, these patients show an association between the reduced motivation subscale and the RER (e.g., 30 ms: *β* = −59.8% EMG_peak_·s^−1^, *P* < 0.001). Only peak force fatigability contributed to fatigue variance, whereas RFD_peak_ did not.

**Conclusions:**

In CKD patients, the neuromuscular fatigability assessed using RFD_peak_ is related to an impairment in motor-unit recruitment or discharge rates, whereas only peak force fatigability was related to fatigue. This suggests that targeting exercise interventions might lessen fatigue and improve quality of life in CKD patients.

Self-reported fatigue is the highest prevalent symptom in chronic kidney disease (CKD) patients (1,2). The prevalence of fatigue in patients with CKD, including those undergoing hemodialysis, ranges between 70% and 80% (2,3). Moreover, the severity of fatigue is reported as similar in both dialysis and non-dialysis-dependent CKD patients (4).

Fatigue is defined as an overwhelming, debilitating, and sustained sense of exhaustion that decreases the ability to carry out daily activities, including the ability to work effectively and to function at one’s usual level in the family or social roles (5). Understanding fatigue symptom is a major research priority because of the effect this has on quality of life and patients’ ability to perform daily activities. Fatigue is independently associated with progression to end-stage renal disease and mortality (6).

CKD is defined as a reduction of the kidney function to less than 60 mL⋅min^−1^ per 1.73 m^2^, or the presence of markers of kidney damage (e.g., hematuria, albuminuria), or abnormalities detected by means of blood sample testing or imaging, lasting for at least 3 months (7). Several studies have been assessed that low renal function, depressive symptoms, low self-reported sleep quality, low albumin level, or restless legs syndrome are associated with severe fatigue (3,4). Self-reported neuromuscular symptoms such as muscle soreness or muscle pain are commonly related to fatigue (8). However, the relationship between objective measures of neuromuscular function and fatigue remains unclear in CKD patients (9).

Neuromuscular fatigability is defined as any decrease in muscle force or power during a standardized task (10,11). Neuromuscular fatigability, unlike subjective fatigue, is reversible with rest (12) and affected by determinants such as the task performed or the force exerted. Although fatigue and neuromuscular fatigability are different, neuromuscular fatigability might represent a contributor to fatigue (13). To date, as recently emphasized by Gollie et al. (9,14), few data regarding neuromuscular fatigability in CKD patients are available.

Neuromuscular fatigability is commonly quantified using maximal voluntary force (MVF) decline during a standardized task (12); however, power and speed or accuracy can be used (13). As recently validated, the peak of the rate of force development (RFD_peak_) is considered a valid estimate to quantify neuromuscular fatigability (15). RFD_peak_ is complementary to MVF assessment during exercise because its performances rely more on fast motor-unit recruitment and high discharge rate, especially in the initial phase of the contraction (16,17).

The aims of this study were therefore to identify determinants of neuromuscular fatigability using EMG and to assess the relationship between neuromuscular fatigability and self-reported fatigue in elderly individuals with CKD. Understanding the relationship between fatigue and fatigability could make it possible to target symptom-management interventions.

## METHODS

### Study organization

Our cross-sectional single-center observational study was part of the Physiopathology of Neuromuscular Function Related to Fatigue in Chronic Renal Disease in the Elderly (PIONEER) (18) project. It was approved by Ethical Committee Est-III of the Nancy University Hospital Centre (no. 20.03.01) and recorded into clinical trial (NCT: 04330807). The study was conducted in accordance with the Declaration of Helsinki. All volunteers received an information letter and provided written informed consent before being enrolled.

### Participants

Participants were enrolled between July 2020 and December 2021 in the “Unité d’Insuffisance Renale AVancée” at Centre Hospitalier Le Mans in Sarthe (France) dedicated to the care of CKD patients. Inclusion criteria for the CKD cohort were age older than 60 yr, estimated glomerular filtration rate (eGFR) below 45 mL·min^−1^ per 1.73 m^2^ (stage 3b-5 not on dialysis) calculated by means of the Chronic Kidney Disease Epidemiology Collaboration equation for at least 3 months, and stable clinical condition, defined as not having been hospitalized in the previous 3 months.

CKD patients were compared with non-CKD participants. Controls were 60 yr or older, with an available serum creatinine measurement within the previous 6 months, and an eGFR above 60 mL·min^−1^ per 1.73 m^2^.

Exclusion criteria were inability to give informed consent, being under guardianship, presence of a neuromuscular disease, known severe cognitive impairment, history of upper limb surgery, estimated life expectancy of less than 3 months, and an acute kidney disease or an expected start of dialysis within 3 months for CKD patients.

### Clinical data

The following information about participants was gathered and analyzed in the study: anthropometric data, eGFR calculated using Chronic Kidney Disease Epidemiology collaboration equation and adjusted to body surface area (CKD-EPI no BSA) using the Haycock method (19), Charlson Comorbidity Index (CCI), diabetes mellitus (yes/no), self-reported sleep satisfaction using a visual analogue scale going from 0 “totally dissatisfied with my sleep quality” to 10 “totally satisfied with my sleep quality,” the last available creatinine level (all participants), and hemoglobin level (only for CKD patients).

### Self-reported fatigue assessment

Before starting the neuromuscular fatigability protocol, fatigue was evaluated by means of two widely used questionnaires in CKD patients (20).

The French version of the Multidimensional Fatigue Inventory–20 items (MFI-20) was used. This questionnaire was initially developed for cancer patients (21) before being more widely used. The MFI-20 questionnaire has been validated in French (22) as it is deemed to have satisfactory internal consistency (better than or equal to those of the English version). Subscales are “general fatigue,” defined as the overall aspect of perceived fatigue; “mental fatigue,” defined as the cognitive aspect of fatigue and despondence; “reduced motivation,” defined as difficulty to imagining and planned pleasant activities; and “reduced activities,” defined as the capacity to physically do something. Items are rated using a 7-point scale on which the participant has to indicate the extent to which the statement applies to her or him, from 1 (“Yes, that is true”) to 7 (“No, that is not true”) (22). The MFI-20 is composed of an equal number of positive/negative questions to avoid acquiescence bias. The higher the score, the greater the degree of fatigue perceived. MFI-20 questionnaire enabled us to gather qualitative data on subjective fatigue, meaning that different aspects of the fatigue were distinguished using the questionnaire subscales (23)

The Functional Assessment of Chronic Illness Therapy–Fatigue (FACIT-F) scale was composed of 13 items and has been shown to have excellent reliability (intraclass correlation [ICC] = 0.90) and satisfactory internal consistency (24). FACIT-F was used with CKD patients (4), and the French translation is validated (25). Items are composed of a 5-point scale, and the participant has to rate the intensity experienced from 0 (“not at all”) to 4 (“very much”) based on a 7-d recall period. A FACIT-F score <40 is considered as a high degree of fatigue, based on the data for nonanemic cancer patients (26). This threshold makes it possible to determine a quantitative fatigue score (26).

### Neuromuscular fatigability assessment

The protocol focused on measuring the isometric force of the dominant finger flexor muscles because this is the muscle group most commonly studied in the clinical setting (27) and is less prone to vascular and neuropathic complications (28). In addition, the neuromuscular fatigability as assessed using the handgrip task can be considered representative to the neuromuscular fatigability obtained in the lower limb muscles (29). The participant sat upright with their elbow bent at a 90° angle close to their chest. The humerus was vertical, and the horizontally placed forearm rested on a height-adjustable support. The nondominant arm rested on the participant’s leg.

**FIGURE 1:**
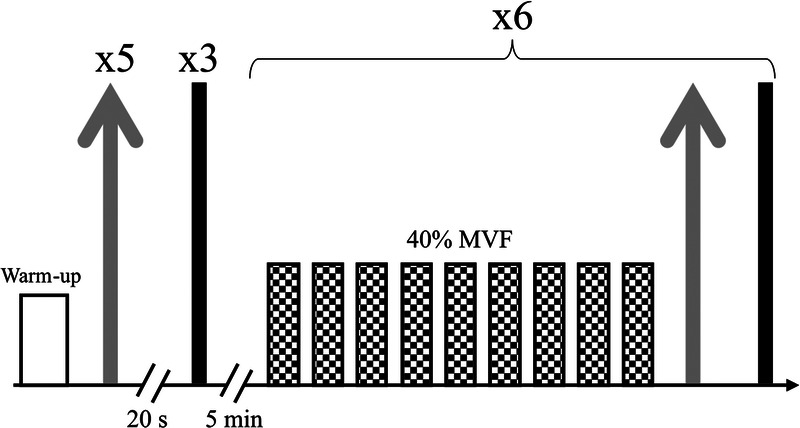
Description of the neuromuscular fatigability protocol. *Gray arrows* represent explosive contractions, *black lines* represent maximal voluntary contractions, and the *gridded boxes* represent submaximal contractions.

The experimental protocol (Fig. 1) was composed of two parts. The first was a warm-up period consisting of dynamic extensions of the fingers. Then five explosive contractions, defined as rapid-force productions, were performed directly followed by a relaxation time with the following instructions: “Grip the dynamometer as fast and as hard as possible and release it immediately afterwards.” Explosive contractions were performed after a 3-s countdown; they lasted 1 s and were interspersed with 20-s rest intervals. Finally, three maximum voluntary contractions given vigorous vocal encouragement were performed after a 3-s countdown to assess the MVF. The following instructions were given: “Grip the dynamometer as hard as you can until I tell you to stop.” Each contraction lasted 5 s with 2-min rest intervals between them and a 5-min break scheduled after the last attempt. As recommended, explosive contractions were performed separately from the maximum voluntary contractions (30). This part of the protocol defined neuromuscular performances at rest.

Second, the fatigability period was studied in accordance with Bigland-Ritchie and Woods (1984). The protocol consisted of nine isometric submaximal contractions at 40% MVF, followed by one explosive contraction and one maximum voluntary contraction (31). These 11 contractions were repeated six times. Each submaximal contraction lasted 3 s and was followed by a 2-s rest.

### EMG acquisition

Surface EMG (sEMG) activity was recorded using Trigno® Wireless Biofeedback System (Delsys Inc., Natick, MA). The sensors used are composed of two pairs of silver bar contacts with 10 mm interelectrode spacing; they have a sampling rate of 1926 Hz, a common-mode rejection ratio of 80 dB, and a 2.4-GHz transmission frequency. Before electrode placement, the skin was shaved and wiped clean with 70° alcohol to reduce impedance. Four electrodes were placed and secured with double side tape and kinesiology taping following the SENIAM recommendation and the anatomical location previously described (32).

### Dynamometry acquisition

The dynamometer used was the K-Force Grip (Kinvent Biomecanique SAS., Montpellier, France) with a sampling rate of 1000 Hz and an accuracy of 100 g. It was fixed to a specially made support that ensured correct placement of the hand. As tested in our laboratory, this device has excellent intrarater reliability (ICC_3.k_ = 0.996, 95% confidence interval [CI] = 0.992–0.998; standard error of measurement = 11.17 N, 95% CI = 9.31–13.02; coefficient of variation = 2.8%) as well as excellent interrater reliability (ICC_2_ = 0.971, 95% CI = 0.917–0.987; standard error of measurement = 17.55 N, 95% CI = 13.80–21.30; coefficient of variation = 3.9%) in isometric MVF measurement.

Direct visual feedback of the force applied to the dynamometer was provided using graphic representation by means of LabVIEW v.19.0.1 (National Instruments Corp., Austin, TX). The required force during the submaximal contractions (40% MVF) was represented as a colored horizontal band (±3%) in the force-time graphic, with an alarm set at ±10%. The direct biofeedback interface provided a visual alert if the participant produced a deleterious pretension force higher than 200*g*.

### Offline data processing

The numeric force signal was filtered by means of a second-order zero-lag low-pass filter at 40 Hz cutoff frequency in Matlab R2018a v9.4 (The MathWorks Inc., Natick, MA). The onset of contractions was determined using a second derivative method, with high reliability for upper limb movements (33), ensuring homogeneity in the force rise. MVF (N) was considered as the peak force. At rest, the highest peak force of the three maximal voluntary contractions was considered for reference. The RFD_peak_ (N·s^−1^) in absolute and normalized with MVF (NRFD_peak_ [% MVF·s^−1^]) was calculated during explosive contractions. The normalization of the RFD reflects the ability of volunteers to develop explosively their own force capacities, whereas absolute measure relies mainly on the MVF (16,17). RFD_peak_ was defined as the peak of the first derivative of the force-time signal. The volunteer’s results were discarded if he or she failed to reach 40% of peak force or decreased in force within 200 ms from the onset of contraction. At rest, the highest RFD_peak_ of the five explosive contractions was considered for reference. During the submaximal contractions at 40% MVF, the integral of the force normalized with MVF was calculated (% MVF·s^−1^) to quantify the participant’s ability to perform muscular tasks.

The sEMG signal was filtered using a fourth-order Butterworth band-pass filter with a 10- to 500-Hz frequency cutoff. The signal-to-noise ratio was estimated in the four sEMG channels during the three maximal voluntary contractions. The electrode with the highest signal-to-noise ratio at MVF was considered for analysis. The sEMG signal was then rectified and smoothed using a zero-lag low-pass filter with 20 Hz cutoff frequency. Each sEMG burst was manually selected as described (34). The smoothed sEMG was used to determine the mean EMG (μV) during maximal voluntary contractions and the rate of EMG rise (RER) during explosive contractions. RER was defined as the slope (∆sEMG/∆time) of the smoothed sEMG signal calculated in time intervals of 0–30, 0–50, and 0–75 ms (RER_0–30_, RER_0–50_, RER_0–75_, respectively) relative to the onset of sEMG activity (35). No additional time interval was considered because it is common for a decrease in sEMG amplitude to occur after ~80–100 ms (35,36). RER was considered in absolute terms (μV·s^−1^) and normalized (NRER) with the peak of the smoothed sEMG signal at MVF (%sEMG_peak_·s^−1^).

Because of the short period of time for explosive contraction (0–300 ms relative to the onset of contraction), the frequency domain was analyzed using a continuous wavelet transform of the raw sEMG (37). The time-frequency domain was considered within the 10- to 500-Hz bandwidth frequency, and a ±500-ms margin of time was conserved for signal transformation to avoid edge effects. Signals were computed using the Morse mother wavelet with *β* = 60 and *γ* = 3 parameters (38), and the complex matrices were made absolute and squared (37). The instantaneous median frequency (i*fmed*) was computed as the frequency value dividing the power spectra in two halves of the same energy. The mean i*fmed* was then calculated for the whole MVC, for each submaximal contraction and within 0–75, 75–150, 150–225, and 225–300 ms time periods relative to the onset of the explosive contractions (36). Although traditional mean and median frequency cannot be used during short periods of time such as explosive contractions, the use of i*fmed* was also motivated as a frequency domain estimate because it was considered to be a better sEMG descriptor of muscle fatigue than traditional mean or median frequency (39) with less variability in isometric tasks (40).

Finally, the fatigability index (FI) was calculated in force (RFD_peak_ and MVF) and sEMG estimates (mean EMG, RER, and i*fmed*) using equation 1. FI was considered as a neuromuscular fatigability estimate because it represents the relative neuromuscular performance capacities at the end of the task compared with rest. A lower value represents a higher relative decrease in performance, consequently higher neuromuscular fatigability.


$$\mathrm{FI}\;\left(\text{\%}\right)=\left(\frac{\text{final value}}{\text{inital value}}\right)100$$ [1]

### Statistical analysis

Data were analyzed using RStudio v.4.0.5 (R Core Team 2021, Vienna, AT). The inclusion period ended before reaching the *a priori* sample size calculated (*n* = 220 for 96% statistical power) (18) because of an underestimation of the outcome (∆8% chosen, ∆11% observed). Continuous data were tested for normality using the Shapiro–Wilk test and displayed as mean and SD, or median and interquartile range (IQR). Student *t*-test was used for normal distribution; otherwise, the Wilcoxon rank sum test was preferred. Qualitative data are shown with percentages and compared using chi-square test. Univariate and multivariate logistic regressions (e.g., to test the outcome CKD vs controls) or linear regressions (e.g., MFI-20: total score) were used to test fatigue determinants. The determinants of neuromuscular fatigability were tested using multilevel models overall and within each group. Random slopes and intercepts were allowed according to time and participants, respectively. All the models were adjusted for age, sex, body mass index (BMI), CCI, and sleep satisfaction. The models for each group were also adjusted for eGFR CKD-EPI no BSA, and CKD models were adjusted for hemoglobin level. Conditions of application of the models were verified using “performance” R-package in testing linearity and homogeneity of variance, collinearity between covariables (with a variance inflation factor of less than 5), influential observations, and normality of residuals. Accordingly, the best type of fitting (linear or gamma) was then chosen, making model interpretation possible, and the exclusion of influential or outlier measures was performed if required.

The influence of neuromuscular fatigability on fatigue was further tested in subsets of participants (both CKD patients and controls) with higher and lower degrees of fatigue (FACIT-F < 40). First, a multiple linear regression with backward deletion method was used (11) to compose the null model. Second, the null model was compared with the null model plus the MVF-FI or the RFD_peak_-FI using ANOVA. Coefficients of determination were compared to quantify the part of the neuromuscular fatigability related to fatigue description. An alpha risk of 5% was chosen.

## RESULTS

### Baseline Data and Self-reported Fatigue

The study initially recruited 161 participants, 51 of whom were excluded for one of the following reasons: receiving antidepressive or antiepileptic treatment (*n* = 22); mild cognitive impairment or neurologic disorder (*n* = 10); chronic pain impairing daily activities (*n* = 5); insufficient time to complete the tests (*n* = 5); unstable clinical condition, including post-COVID 19 fatigue (*n* = 5); dropout from test (*n* = 3); and severely impaired visual acuity (*n* = 1). Eight other participants were excluded because of inconsistent explosive contractions (five at rest and three during the fatigability period). The individuals excluded were older than those included (76 [16_IQR_] vs 70 [11_IQR_], *P* = 0.024, respectively) but showed no other difference (see Supplemental Table 1, Supplemental Digital Content 1, Comparison of data between excluded individuals and those included, http://links.lww.com/MSS/C754). Table 1 shows the baseline data of the 102 participants considered, of whom 45 were CKD patients and 57 controls.

**TABLE 1:** Baseline data

	All	CKD	Controls	*P*
*n*	102	45	57	
Age (yr), median (IQR)	69 (9)	70 (11)	67 (9)	**0.038**
Sex (females), *n* (%)	39 (38.2%)	12 (26.7%)	27 (47.4%)	**0.033**
BMI (kg·m^−2^)	27.6 (6.2)	27.8 (5.4)	27.1 (6.7)	0.272
≥30 (%)	30.4%	28.9%	31.6%	0.672
≥35 (%)	8.8%	11.1%	7.0%	0.469
Hemoglobin (mg·dL^−1^), median (IQR)	13.2 (3.0)	12.8 (2.4)	13.7 (2.5)*^a^*	0.119
Creatinine (mg·dL^−1^)		2.05 (2.11)	1.01 (1.23)	**–**
eGFR CKD-EPI (mL·min^−1^ per 1.73 m^2^)	**–**	29 (18)	88 (13)	**–**
eGFR CKD-EPI no BSA (mL⋅min^−1^)	**–**	25 (18)	78 (23)	**–**
CKD stages in patients, *n* (%)				
Stage 3b		21 (46.7%)		
Stage 4		19 (42.2%)		
Stage 5		5 (11.1%)		
CCI (score)	4 (3)	6 (3)	3 (2)	**<0.001**
Diabetes mellitus (% yes)	39.2%	40.0%	38.6%	0.885
Sleep satisfaction (score)	8 (3)	7 (3)	8 (2)	0.164
Blood pressure (mm Hg), median (IQR)				
Systol	140 (22)	140 (20)	142 (29)	0.887
Diastol	74 (12)	75 (10)	76 (11)	0.501
Heart rate at rest (bpm), median (IQR)	70 (15)	70 (16)	68 (16)	0.355
Dominant arm (right-handed), *n* (%)	99 (97.1%)	44 (97.8%)	55 (96.5%)	0.999

Bold values are statistically significant for *P* < 0.05.

*^a^n* = 9 (16%) of missing data for hemoglobin level in controls.

eGFR CKD-EPI, estimated glomerular filtration rate using the Chronic Kidney Disease Epidemiology collaboration equation; no BSA, eGFR CKD-EPI adjusted for body surface area using the Haycock method (19).

In a nonadjusted model, there was a 2.9-fold higher risk that patients would suffer a higher degree of fatigue (on the basis of FACIT-F < 40) than controls and show higher fatigue in all the fatigue subscales, except for reduced motivation (Table 2). When adjusted for age, sex, BMI, sleep satisfaction, and CCI, CKD patients reported higher fatigue than controls in the MFI-20 total score and its mental and reduced activity subscales, but with FACIT-F < 40 cutoff, their risk of being more fatigued was not significantly higher. In CKD patients, no relationship was found between the eGFR and the fatigue subscales in univariate and adjusted on age, sex, BMI, CCI, sleep satisfaction, and hemoglobin. As shown in Supplemental Figure 1 (see Supplemental Digital Content 2, Relation between MFI-20 and FACIT-F total score, http://links.lww.com/MSS/C755), there is high agreement between MFI-20 and FACIT-F for all degrees of fatigue.

**TABLE 2:** Comparison of fatigue using qualitative and quantitative approaches between CKD and controls

				Model					
	Theoretical			Unadjusted			Adjusted*^a^*		
	Min–Max	CKD	Controls	*β*	95% CI	∆ %	*β*	95% CI	∆ %
Qualitative									
MFI-20 subscales, mean (SD)									
General	9–63	33.2 (10.9)	24.0 (12.0)	**9.21**	**4.65 to 13.77**	17.1 %	5.28	−0.62 to 11.18	N.S.
Mental	6–42	15.4 (6.8)	11.9 (7.0)	**3.52**	**0.80 to 6.24**	9.8 %	**4.57**	**0.80 to 8.33**	12.7 %
Reduced activity	3–21	10.6 (4.9)	6.8 (4.4)	**3.80**	**1.96 to 5.64**	21.1 %	**3.36**	**0.85 to 5.87**	18.7 %
Reduced motivation	2–14	5.6 (2.9)	4.5 (2.9)	1.07	−0.07 to 2.21	N.S.	0.18	−1.72 to 1.37	N.S.
Quantitative									
Total score, mean (SD)									
MFI-20	20–140	64.8 (20.9)	47.2 (22.0)	**17.59**	**9.09 to 26.10**	14.7 %	**13.03**	**1.69 to 24.36**	10.9 %
FACIT-F	0–52	38.6 (6.7)	44.0 (8.0)	−**5.39**	−**8.33 to** −**2.45**	10.4%	−2.69	−6.50 to 1.11	N.S.
FACIT-F < 40, *n* (%)*^b^*	Yes/No	23 (51.1%)	15 (26.3%)	**2.93**	**1.29 to 6.84**	–	1.27	0.37 to 4.24	–

*β* represents the difference in fatigue score for CKD patients compared with the control group and expressed in relation to the theoretical maximum, calculated using *β*/theoretical range × 100 (∆ %). Bold values are statistically significant for *P* < 0.05.

Higher MFI-20 and lower FACIT-F scores represent greater fatigue.

*^a^*Adjusted for age, sex, BMI, sleep satisfaction, and CCI.

*^b^*Because of the dichotomic outcome, a logistic regression model was performed, and the result was expressed as an odds ratio rather than *β*.

N.S., nonsignificant.

### Association between CKD and Neuromuscular Performance

**FIGURE 2:**
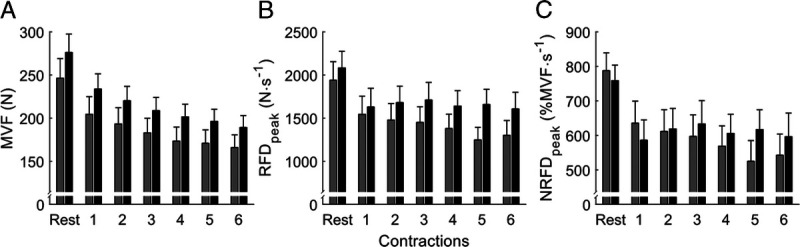
Evolution of the MVF (A), RFD (B), and NRFD (C) during the fatigability protocol. *Gray bars* represent CKD patients, and *black bars* represent controls with the 95% CI.

CKD patients had lower MVF than controls, adjusted for age, sex, BMI, CCI, and sleep satisfaction. Despite lower MVF, CKD patients showed similar decrement during the fatigability protocol (Fig. 2, Table 3). Nevertheless, the NRFD_peak_ shows a group–time interaction in showing 2.1-fold higher rate of decrease during the fatigability protocol compared with controls (Fig. 2). Consequently, CKD patients had reduced capacity to perform the muscular tasks required during the 40% MVF submaximal contractions, which is evidenced in the group–time interaction (*β* = −0.1% MVF·s^−1^, *P* = 0.014; Supplemental Table 2, Supplemental Digital Content 1, Determinants of the neuromuscular estimates during the submaximal contraction at 40% MVF, http://links.lww.com/MSS/C754). The eGFR was not associated with muscular performance in the CKD patients in our cohort.

**TABLE 3:** Determinants of force estimates during the fatigability protocol

	Model^*a*^								
	Overall			CKD			Control		
	*β*	95% CI	*P*	*β*	95% CI	*P*	*β*	95% CI	*P*
MVF (N)									
Contractions (time)	−12.622	−14.347 to −10.897	**<0.001**	−12.027	−13.840 to −10.213	**<0.001**	−12.590	−14.274 to −10.906	**<0.001**
Group (CKD vs Controls)	−19.234	−37.954 to −0.514	**0.044**	–			–		
Contraction–group	0.408	−2.118 to 2.934	0.751	–			–		
MFI-20 domains (score)									
General	0.368	−0.227 to 0.963	0.225	1.482	0.620 to 2.343	**<0.001**	−0.249	−1.172 to 0.675	0.598
Mental	−2.089	−2.971 to −1.206	**<0.001**	−1.292	−2.577 to −0.008	**0.049**	−2.295	−3.647 to −0.942	**<0.001**
Motivation	−0.096	−2.019 to 1.827	0.922	−1.811	−4.556 to 0.933	0.196	0.113	−2.581 to 2.808	0.934
Activity	0.106	−1.191 to 1.403	0.873	−2.884	−4.737 to −1.031	**0.002**	2.266	0.432 to 4.100	**0.016**
RFD_peak_ (N·s^−1^)									
Contractions (time)	−46.824	−73.146 to −20.502	**<0.001**	−60.449	−85.566 to −35.333	**<0.001**	−43.076	−69.538 to −16.614	**0.001**
Group (CKD vs controls)	−97.230	−347.978 to 153.518	0.447	–			–		
Contraction–group	−33.818	−72.055 to 4.419	0.083	–			–		
MFI-20 domains (score)									
General	6.722	−1.954 to 15.398	0.129	16.151	6.590 to 25.711	**<0.001**	−4.331	−18.819 to 10.156	0.558
Mental	−20.587	−33.501 to −7.673	**0.002**	−26.195	−40.573 to −11.816	**<0.001**	−13.976	−35.373 to 7.420	0.**200**
Motivation	−12.777	−40.507 to 14.953	0.366	−28.602	−58.440 to 1.267	0.060	18.451	−22.733 to 59.635	0.380
Activity	−8.811	−27.650 to 10.028	0.359	−8.620	−29.907 to 12.668	0.427	−2.847	−31.329 to 25.636	0.845
NRFD_peak_ (% MVF·s^−1^)									
Contractions (time)	−13.912	−23.564 to −4.259	**0.005**	−29.996	−40.800 to −19.191	**<0.001**	−14.033	−23.737 to −4.329	**0.005**
Group (CKD vs controls)	78.960	−11.174 to 169.095	0.086	–			–		
Contraction–group	−16.703	−31.138 to −2.267	**0.024**	–			–		
MFI-20 domains (score)									
General	−3.337	−8.105 to 1.431	0.171	0.680	−3.836 to 5.195	0.768	1.446	−3.657 to 6.548	0.579
Mental	−5.177	−15.347 to 4.993	0.319	−6.497	−13.069 to 0.076	0.053	−1.935	−9.458 to 5.589	0.614
Motivation	−2.555	−9.433 to 4.322	0.467	−8.874	−23.430 to 5.682	0.232	9.412	−5.200 to 24.023	0.207
Activity	−3.337	−8.105 to 1.431	0.171	1.680	−7.877 to 11.237	0.730	−2.923	−12.972 to 7.126	0.569

*^a^*All models were adjusted for age, sex, BMI, CCI, and sleep satisfaction. CKD and control models were also adjusted for eGFR CKD-EPI no BSA, and CKD models were adjusted for hemoglobin level.

eGFR CKD-EPI no BSA, estimated glomerular filtration rate using the Chronic Kidney Disease Epidemiology collaboration equation adjusted for body surface area using the Haycock method (19); (N)RFD_peak_, peak of the (normalized) rate of force development. A higher MFI-20 score represents greater fatigue.

During the submaximal contractions at 40% MVF, the mean EMG increased with time (*β* = 0.3% EMG_peak_, *P* < 0.001) and the i*fmed* decreased (*β* = −0.2 Hz, *P* < 0.001), with no differences in sEMG parameters between groups (see Supplemental Table 2, Supplemental Digital Content 1, Determinants of the neuromuscular estimates during the submaximal contraction at 40% MVF, http://links.lww.com/MSS/C754). During the maximal voluntary contractions, mean EMG decreased in absolute terms (*β* = −5.2 μV, *P* < 0.001) and when normalized with the FI (*β* = −2.2 %, *P* < 0.001), as did the i*fmed* (*β* = −1.2 Hz, *P* = 0.005), with no difference between CKD patients and controls (see Supplemental Table 3, Supplemental Digital Content 1, Determinant of the sEMG parameters during the maximal voluntary contractions of the fatigability protocol, http://links.lww.com/MSS/C754). However, despite there being no significant interaction in sEMG between groups and time during explosive contractions, CKD patients decreased their NRER with a 1.9- to 3.1-fold higher rate compared with controls (Table 4), with significant decreased in RER with time only in CKD (RER_0–30_: *β* = −113.5 μV·s^−1^, *P* = 0.012 in CKD patients, *β* = −71.7 μV·s^−1^, *P* = 0.085 in controls; RER_0–50_: *β* = −121.2 μV·s^−1^, *P* = 0.016 in CKD patients, *β* = −48.5 μV·s^−1^, *P* = 0.196 in controls; RER_0–75_: *β* = −102.4 μV·s^−1^, *P* = 0.006 in CKD patients, *β* = −40.2 μV·s^−1^, *P* = 0.166 in controls; Supplemental Table 4, Supplemental Digital Content 1, Determinant of the absolute RER during the fatigability protocol, http://links.lww.com/MSS/C754). The i*fmed* during explosive contractions showed a similar evolution with time between groups when considering all the time periods together (see Supplemental Table 5, Supplemental Digital Content 1, Determinant of the i*fmed* during explosive contractions considering all the different time period during the fatigability protocol, http://links.lww.com/MSS/C754) and was significantly decreased with time during the maximal voluntary contractions only in CKD patients (see Supplemental Table 3, Supplemental Digital Content 1, Determinant of the sEMG parameters during the maximal voluntary contractions of the fatigability protocol, http://links.lww.com/MSS/C754). No independent relationship between eGFR and sEMG parameters was noticed in our cohort.

**TABLE 4:** Determinants of the normalized RER in different time periods during the fatigability protocol

	Model^*a*^								
	Overall			CKD			Controls		
	*β*	95% CI	*P*	*β*	95% CI	*P*	*β*	95% CI	*P*
NRER 0–30 ms (%EMG_peak_·s^−1^)									
Contractions (time)	−6.803	−20.332 to 6.726	0.325	−17.386	−37.743 to −2.971	0.101	−9.274	−25.811 to 7.263	0.272
Group (CKD vs Controls)	−67.695	−208.243 to 72.853	0.345	–			–		
Contraction × Group	−7.320	−26.722 to 12.082	0.460	–			–		
MFI-20 domains (score)									
General	−0.949	−6.173 to 4.276	0.722	−2.041	−11.636 to 7.555	0.680	−1.558	−13.216 to 10.099	0.794
Mental	−2.924	−10.839 to 4.992	0.469	10.738	−3.313 to 24.788	0.144	−11.383	−28.453 to 5.688	0.198
Motivation	−20.754	−37.844 to −3.664	**0.018**	−59.764	−90.891 to −28.636	**<0.001**	4.376	−29.787 to 38.539	0.803
Activity	13.323	1.313 to 25.333	**0.030**	11.657	−8.912 to 32.226	0.275	16.583	−6.477 to 39.643	0.165
NRER 0–50 ms (%EMG_peak_·s^−1^)									
Contractions (time)	−5.292	−23.210 to 12.625	0.563	−15.226	−38.307 to 7.862	0.203	−5.292	−21.753 to 11.168	0.527
Group (CKD vs Controls)	−76.549	−252.917 to 99.819	0.395	–					
Contraction × Group	−11.048	−38.024 to 15.927	0.422	–					
MFI-20 domains (score)									
General	−1.106	−8.225 to 6.013	0.761	−2.318	−12.376 to 7.741	0.655	0.672	−11.353 to 12.696	0.913
Mental	−6.436	−17.065 to 4.193	0.236	8.215	−6.513 to 22.943	0.283	−14.136	−31.744 to 3.472	0.122
Motivation	−14.804	−38.026 to 8.419	0.212	−54.760	−87.389 to −22.130	**0.002**	7.003	−28.236 to 42.241	0.698
Activity	13.685	−1.833 to 29.203	0.084	11.303	−10.258 to 32.864	0.312	13.494	−10.292 to 37.280	0.272
NRER 0–75 ms (%EMG_peak_·s^−1^)									
Contractions (time)	−4.848	−18.490 to 8.794	0.488	−14.774	−32.175 to 2.627	0.103	−4.848	−17.166 to 7.469	0.444
Group (CKD vs Controls)	−26.300	−153.596 to 100.995	0.686	–			–		
Contraction × Group	−10.616	−31.155 to 9.923	0.314	–			–		
MFI-20 domains (score)									
General	0.253	−4.915 to 5.422	0.924	−0.710	−9.089 to 7.670	0.869	1.671	−6.307 to 9.649	0.683
Mental	−5.898	−13.615 to 1.819	0.138	3.962	−8.308 to 16.232	0.531	−11.776	−23.459 to 0.001	0.054
Motivation	−7.377	−24.238 to 9.483	0.391	−38.345	−65.529 to −11.162	**0.009**	10.273	−13.108 to 33.654	0.394
Activity	8. 471	−2.796 to 19.737	0.144	6.892	−11.071 to 24.854	0.458	8.617	−7.165 to 24.399	0.290

*^a^*All the model were adjusted for age, sex, BMI, CCI and sleep satisfaction. CKD and control models were also adjusted for eGFR CKD-EPI no BSA, and CKD models were adjusted for hemoglobin level.

eGFR CKD-EPI no BSA, estimated glomerular filtration rate using the Chronic Kidney Disease Epidemiology collaboration equation adjusted for body surface area using the Haycock method (19); NRER, rate of EMG rise calculated on a normalized signal with the individual peak of EMG at MVF. A higher MFI-20 score represents greater fatigue.

### Association between Fatigue Subscales and Neuromuscular Performance (Qualitative)

#### MFI-20: general fatigue

In the models in CKD patients (Table 3), the higher the general fatigue, the higher the MVF and RFD_peak_.

#### MFI-20: mental fatigue

Mental fatigue was associated with a lower RFD_peak_ in CKD patients and a lower MVF in both CKD patients and controls (Table 2) and with a higher i*fmed* during the submaximal contractions (*β* = 0.8 Hz, *P* = 0.045; Supplemental Table 2, Supplemental Digital Content 1, Determinants of the neuromuscular estimates during the submaximal contraction at 40% MVF, http://links.lww.com/MSS/C754). During maximal voluntary contractions and only in controls, this subscale was associated with a higher i*fmed* (*β* = 0.9 Hz, *P* = 0.020; Supplemental Table 3, Supplemental Digital Content 1, Determinant of the sEMG parameters during the maximal voluntary contractions of the fatigability protocol, http://links.lww.com/MSS/C754).

#### MFI-20: reduced motivation

Reduced motivation fatigue was associated with a lower RER for CKD patients in almost all of the time periods considered (RER_0–30_ = *β* = −137.6 μV·s^−1^, *P* = 0.042; RER_0–50_ = *β* = −151.6 μV·s^−1^, *P* = 0.038; Supplemental Table 4, Supplemental Digital Content 1, Determinant of the absolute RER during the fatigability protocol, http://links.lww.com/MSS/C754) and a lower NRER_0–30_, NRER_0–50_, and NRER_0–75_ (Table 4).

#### MFI-20: reduced activity

The reduced activity domain was significantly associated with a lower MVF in CKD patients. On the contrary, this MFI-20 subscale was associated with higher MVF in controls (Table 3) and a higher mean EMG normalized with the FI (*β* = 2.8 %, *P* = 0.017; Supplemental Table 3, Supplemental Digital Content 1, Determinant of the sEMG parameters during the maximal voluntary contractions of the fatigability protocol, http://links.lww.com/MSS/C754).

### Association between Degree of Fatigue and Neuromuscular Performance (Quantitative)

**FIGURE 3:**
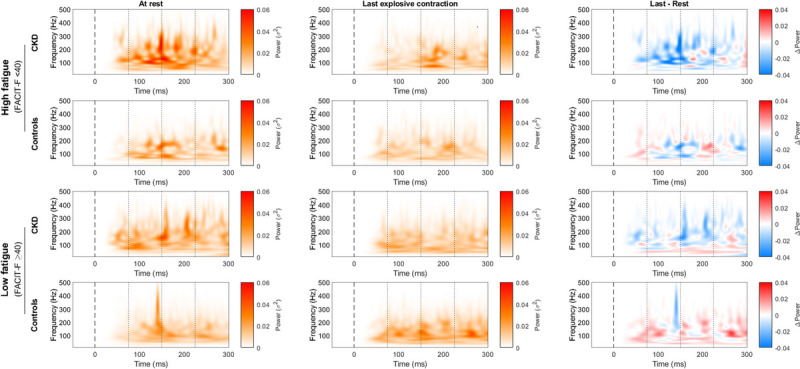
Continuous wavelet transform analysis of the first and last explosive contractions within groups and degrees of fatigue. Onsets of contractions are symbolized with a *dashed line* at time 0, and the *dotted lines* represent the time period considered for i*fmed* analysis. The left panel represents the first 300 ms from the onset of the explosive contractions at rest, the middle panel shows the last explosive contraction, and the right panel maps differences (*blue* represents a higher decrease in energy with fatigability protocol).

All force and sEMG estimates were normalized with rest capacities using the FI (equation 1) and analyzed with respect to degree of fatigue. CKD patients with a high level of fatigue had lower RFD_peak_-FI than controls (*P* = 0.018 and *P* = 0.049 adjusted for age and sex). This means that the higher neuromuscular fatigability noticed in CKD patients (Table 3) is mainly underlined to those with a high degree of fatigue. No differences were noticed in the FI of the MVF, RER, or in the FI of the mean EMG during maximal voluntary contractions. However, the i*fmed*’s FI was significantly reduced within the 75- to 150-ms time period relative to the onset of contractions (*P* = 0.047 and *P* = 0.008 adjusted for age and sex; Supplemental Figure 2, Supplemental Digital Content 2, Comparison of i*fmed* between CKD patients and controls in the high- and low-fatigue groups within different time periods, http://links.lww.com/MSS/C755). The continuous wavelet transform analysis supported this finding and showed a drop in sEMG energy in the high-frequency domain (150–300 Hz) between ~75 and 180 ms in the high-fatigue group of CKD patients (Fig. 3). In these patients, the decrease in high-frequency energy with the fatigability protocol, at the initiation of the explosive contractions, reduced the FI of the i*fmed* (see Supplemental Figure 2, Supplemental Digital Content 2, Comparison of i*fmed* between CKD patients and controls in the high- and low-fatigue groups within different time periods, http://links.lww.com/MSS/C755). The continuous wavelet transform analysis also highlighted a systematic decrease in energy content during the fatigability protocol. CKD patients and controls in the low-fatigue group showed a decrease in high frequencies and an increase in low frequencies (25–75 Hz) with the task, which was less marked in the high-fatigue control group and absent in the high-fatigue CKD group.

### Influence of Neuromuscular Fatigability on Self-reported Fatigue Description

Table 5 shows the significant independent clinical variables for each fatigue subscale and the effect of adding FI estimates. Neuromuscular fatigability, assessed with MVF-FI, significantly improved fatigue description in CKD patients in the groups with lower degrees of fatigue, most notably for the MFI-20 total score. In these patients, the improvement of the MFI-20 subscale description was 7.0%, 10.4%, 11.1%, and 14.7% for the reduced motivation, reduced activity, general fatigue, and mental fatigue, respectively. In the models with MVF-FI, there was a mean 12.2% improvement in fatigue description. Conversely, adding RFD_peak_-FI to the models did not improve fatigue description in any group.

**TABLE 5:**
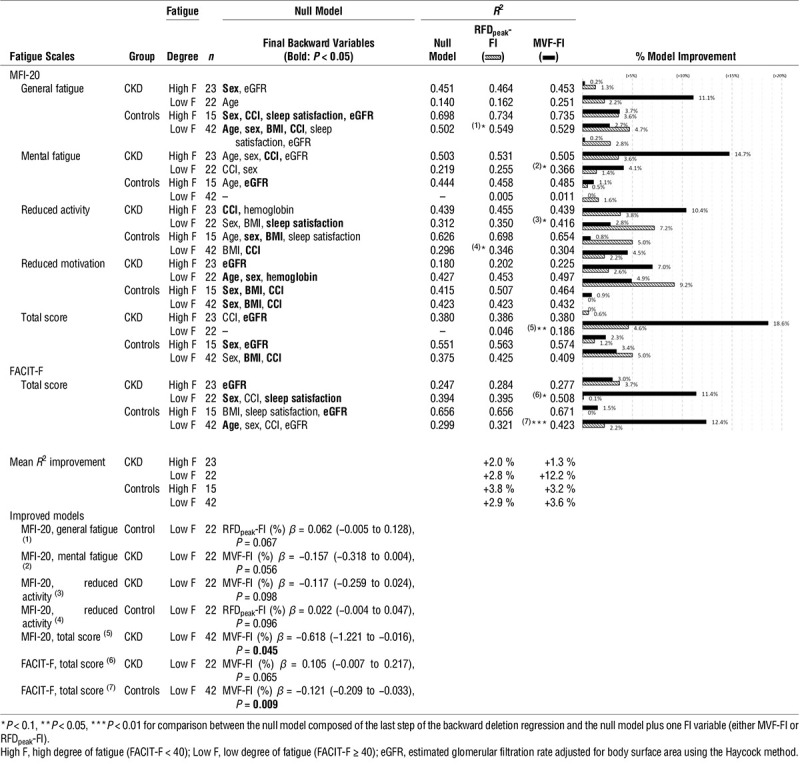
Identification of the population in which neuromuscular fatigability improved fatigue symptom description using model comparison

In models with CKD patients in the low-fatigue group where MVF-FI improved fatigue description, if causal relationship would be shown, an increase of 10% in MVF-FI corresponding to a reduction of 10% in neuromuscular fatigability was associated with a 6.2% reduction in the MFI-20 total score (*β* = −6.2/120 points, *P* = 0.045).

## DISCUSSION

The present study sought to provide new insights into the relationship between neuromuscular fatigability and self-reported fatigue, analyzing a cohort of non-dialysis-dependent elderly CKD patients compared with a control group. The study found that CKD patients had a 2.9 higher risk of manifesting a high degree of fatigue compared with controls, and that the Reduced Activity fatigue subscale was the one most impacted. CKD patients demonstrated higher neuromuscular fatigability assessed using NRFD_peak_, especially those with higher degrees of fatigue. Neuromuscular fatigability in CKD patients was found to be related to a higher decrease in RER over time, concurrent with a drop in i*fmed* at initiation of contraction. The reduction in RER with exercise was most affected in CKD patients with higher scores on the reduced motivation subscale. Neuromuscular fatigability assessed using MVF-FI contributed to 12.2% of fatigue variability in CKD patients with a low degree of fatigue. Thus, this study is the first to quantify a possible association between neuromuscular fatigability and self-reported fatigue in elderly CKD patients.

### The fatigue subscales were differently affected by CKD

Previous studies reported a higher prevalence of fatigue in CKD patients compared with age-matched controls (1,2). Our results are in accordance with these findings and show a higher prevalence and a higher degree of fatigue in CKD patients. Our FACIT-F score (38.6 ± 6.7) is in accordance with Macdonald and Jhamb, which showed a mean score of 35.7 ± 11.8 (41) and 34.3 ± 11.3 (4), respectively. Interestingly, Jhamb reported the same level of fatigue in dialysis patients. This mean level of fatigue is similar to those observed in other pathologies such as cardiovascular diseases (35.0 ± 11.0 [42]), cancer (36.0 ± 12.1 [43]), stroke (38.1 ± 9.6 [43]), and human immunodeficiency virus (34.0 ± 12.6 [43]). The mean FACIT-F score (43.9 ± 8.0) of our control group is similar to those in other studies: for instance, Cella found a mean FACIT-F score of 43.6 ± 9.4 in 1010 U.S. adults (26), and Montan reported a mean FACIT-F of 43.5 ± 8.3 in 2426 German adults (24). The greater risk of CKD patients of being considered in the high degree of fatigue did not remain statistically significant when adjusted for covariates, most likely because a significant proportion of fatigue severity is explained by the trans-diagnostic factors (44). The agreement between MFI-20 and FACIT-F supports consistency of both measures in this population.

The MFI-20 subscales are difficult to compare with those in other studies because the validated French version has a reduced number of domains (merging the General and Physical domain) and lists some items in different domains (22). However, the qualitative approach to fatigue assessment is maintained (23). MFI-20 total score and mental and reduced activity fatigue subscales were significantly and independently related to CKD after adjustment for age, sex, BMI, sleep satisfaction, and comorbidities. This probably reflects the sedentary lifestyle and cognitive impairments frequently reported in CKD patients. Interestingly, CKD seems to be independently related to specific aspects of fatigue, i.e., mental and reduced activity subscales, but not with others, namely, general and reduced motivation. Determinants of fatigue belong to a pool of interconnected symptoms, supporting the approach using symptom clusters in CKD patients (8).

### CKD patients show high neuromuscular fatigability when assessed using NRFD_peak_

Non-dialysis-dependent CKD is commonly associated with a reduction in muscle mass and function. Surprisingly, very few studies have investigated neuromuscular function during a standardized task (9). Heiwe et al. (14) performed a time-to-task failure test of quadriceps muscles in CKD patients. No difference in neuromuscular fatigability with respect to controls was shown. Our results show that the decline in MVF is equivalent to the one found for controls, in keeping with the results of Heiwe et al. Nevertheless, the force–fatigability relationship implies that individuals with lower MVF at rest might have a limited reduction of MVF during exercise compared with those with higher MVF. Despite a lower MVF in CKD patients, the present results demonstrate the same reduction in muscle performance in these patients as in controls, suggesting possibly greater neuromuscular fatigability. Moreover, our study shows that CKD patients had higher neuromuscular fatigability doing explosive contractions using NRFD_peak_, with a two times higher rate of decline during the standardized task. Our result is in accordance with previous studies showing that the use of RFD_peak_ may be more sensitive to quantify neuromuscular fatigability than MVF (15). This different result presumably depends on the parameter used and is related to physiological determinants in CKD patients.

### Impairment in neural drive is associated with CKD

If CKD patients, especially those manifesting higher degrees of fatigue, are not able to maintain motor-unit recruitment and discharge rates during a fatiguing task, they will consequently reduce their RFD_peak_, whereas MVF will be less affected. In fact, the ability to produce force within a very short period of time is dependent on the rapid recruitment of motoneurons with high discharge rates (35) because MVF is more dependent on structural muscle properties (17). This is in accordance with our results, showing both a higher reduction in NRFD_peak_ and a significant reduction with time in RER in CKD patients (from a 1.6 to 3.1 times higher rate of decrease than in controls, depending on the time period or normalization). This decreased speed of recruitment and discharge rates of motoneurons with neuromuscular fatigability may lead to functional impairment. This hypothesis is in accordance with Gollie, which shows a significant correlation between sit-to-stand test and RFD in CKD stage 3b to stage 4 patients (45). The relevant reduction in motoneuron output is supported by the spectral analysis of the sEMG content.

The shift from high to low frequency was expected as it represents a common neural adaptation to a task. Because fast and slow motoneurons induce different shapes and velocities of motor-unit action potential, the frequency content of sEMG changes can be used as a muscle fatigue estimate (46). Several factors influence the spectral properties of the sEMG, such as skinfold thickness, fiber diameter, and the distance between the motoneuron end plate and the sEMG electrode, and its interpretation remains controversial (47). However, because we performed an analysis of the change with fatigability task of the spectral parameters, rather than a crude comparison between individuals, we consider these measures to be valid indicators of sEMG adaptation to neuromuscular fatigability (37,46).

Contrary to other patient groups, CKD patients with a high level of fatigue have possibly reduced capacities to recruit and discharge motor units, at both high and low frequency, at the initiation of explosive contractions during the fatigability protocol. This supports the higher rate of decrease in RER and the drop in i*fmed* within 75–150 ms from the onset of explosive contractions (see Supplemental Figure 2, Supplemental Digital Content 2, Comparison of i*fmed* between CKD patients and controls in the high- and low-fatigue groups within different time periods, http://links.lww.com/MSS/C755) compared with controls. This reduction of i*fmed* is also shown in the multilevel models where only CKD patients reduced the i*fmed* during maximal voluntary contractions. Different hypotheses can explain the different spectral characteristics and the voluntary activation failure of the muscles in these patients. The proportion of fiber types (fast and slow twitches) affects neuromuscular fatigability development (48) and modifies sEMG frequency (46). However, the literature reports no differences in elderly CKD patients and controls regarding muscle fiber type proportion (14). Our study did not assess fat mass, but BMI and the prevalence of obesity were similar between groups, and we consider that the thickness of the subcutaneous layers is not the main reason for the different spectral characteristics. The reduction in the intracellular pH induced by H^+^ accumulation seems to induce a modification in muscle action potential, leading to reductions in both nerve conduction velocity and sEMG frequency (46). CKD patients stages 3b–5 are already known to have a reduced nerve conduction velocity because of uremic toxins and Na/K-ATPase dysfunction (28). In addition, they are more prone to develop muscle acidosis at matched absolute exercise intensity than controls, which contributes to neuromuscular fatigability (6,41). This could explain the reduction in i*fmed* in high-fatigue CKD patients, but not the higher reduction in sEMG rise. The drop in RER during exercise in CKD patients suggests the existence of a possible central limitation in muscle activation (10).

Brownstein and colleagues recently found an increase in sEMG during cycling in fatigued cancer patients. The authors concluded that the increase in motoneuron activity was a compensatory mechanism of peripheral fatigue (49). Although a different methodology was used, the present results show no interaction in sEMG between time and groups during submaximal contractions (see Supplemental Table 2, Supplemental Digital Content 1, Determinants of the neuromuscular estimates during the submaximal contraction at 40% MVF, http://links.lww.com/MSS/C754) or maximal voluntary ones (see Supplementary Table 3, Supplemental Digital Content 1, Determinant of the sEMG parameters during the maximal voluntary contractions of the fatigability protocol, http://links.lww.com/MSS/C754), suggesting no adaptation of motoneuron output to peripheral impairment. The present study did not allow us to provide an explanation for reduced neural drive in CKD patients. It was previously shown that group III and group IV muscle afferents, which are sensitive to metabolic stimuli (50), depress motor cortical cells and inhibit the homonymous α-motoneurons (51). Because CKD patients are more prone to intramuscular potassium disruption and lactate and H^+^ production (41), it can be hypothesized that the small diameter afferents (groups III and IV) could be overexpressed and that this leads to reduced motoneuron output (50). These results suggest that the neuromuscular fatigability assessed using RFD_peak_ in CKD patients is induced by neural limitations, possibly manifested as an impaired motor-unit recruitment and discharge rates during tasks.

### MVF significantly improves fatigue description in CKD patients with low degree of fatigue

A better understanding of the relationship between neuromuscular fatigability and self-reported fatigue is needed to allow us to target therapeutic approaches (9,13). No matter the time period used or normalization, only in CKD patients was there a significant relationship between the RER and the reduced motivation subscale. Reduced motivation is defined as difficulty to imagining and planned pleasant activities (see Supplemental Figure 3, Supplemental Digital Content 2, Comparison of the MFI-20 items between CKD patients and controls, http://links.lww.com/MSS/C755). There would appear to be no reason explaining a causation relationship, but perhaps they share common mechanisms. Although participants with depressive symptoms were excluded, the possibility of subtle undiagnosed depression should be kept in mind.

The present study shows that neuromuscular fatigability assessed using MVF is an independent predictor of self-reported fatigue in CKD patients and in controls. If a causal relationship were to be confirmed on a larger scale, a 10% improvement in neuromuscular fatigability would result in 6 % improvement in self-reported fatigue assessed with the MFI-20 in CKD patients. However, in CKD patients with high degrees of fatigue, adding neuromuscular fatigability does not improve their fatigue description. Despite low subsample size, we suggest that these patients have a central limitation at rest during maximal contraction, leading to reduced MVF considered as reference in the submaximal task (10). Thus, this discrepancy between CKD patients with low and high fatigue could be explained by a reduced submaximal load calculated in patients with higher degree of fatigue. Interpolated twitch method could make it possible to overcome central limitations in muscular efference and give a better estimate of maximal force (10). The RFD_peak_ estimate using the FI is lower in CKD patients than in controls with regard to the high degree of fatigue, but the regression models show that it did not remain related to the symptom of fatigue. This can be explained by the fact that MVF reflects muscle mass and function, which results in daily-life impairment. Conversely, RFD_peak_ is more closely related to fast motor-unit recruitment and high discharge rate, and its effect on fatigue appears less evident. On the other hand, because neuromuscular fatigability assessed using MVF is related to self-reported fatigue in CKD patients with low degrees of fatigue, this tends to support the use exercise interventions.

### Limitations

The sample was relatively small and the study was cross-sectional. The use of voluntary contractions to assess MVF may be a limitation, and the perceived fatigability was not assessed. Twitch interpolation technique was not used in our study because we hypothesized that the pain induced would reduce the number of participants, leading to a nonrepresentative cohort. Finger flexor muscles are not considered as a functional muscle group. However, the very high prevalence of peripheral neuropathy (52), together with recommendations in neurophysiological testing in these patients (28), led the authors to test upper limbs, for which the handgrip task is the most widely used modality in CKD patients (27). In addition, albeit unclear, it seems that neuromuscular fatigability and its etiology might be interrelated between muscle groups and therefore representative of the individual neuromuscular fatigability (29). Although spontaneous physical activity was not recorded, it seems not to significantly affect neuromuscular fatigability in the elderly (53). However, our study has the advantage of novelty and of using sEMG with high-frequency force signal analysis together with the most widely used and best validated fatigue scales in elderly patients with advanced CKD.

## CONCLUSIONS

To the best of our knowledge, this study is the first to shed light on the determinants of neuromuscular fatigability in non-dialysis-dependent CKD patients using high-frequency force signal analysis and sEMG technique. The study found an association between neuromuscular fatigability and self-reported fatigue in CKD patients. Furthermore, CKD patients showed a greater decrease in RER during a fatiguing task than controls, and this was augmented by fatigue. Nevertheless, the association between neuromuscular fatigability and self-reported fatigue suggests that exercise interventions such as adapted physical activity might mitigate fatigue and improve quality of life in CKD patients. Further studies are required to confirm these results using interpolated twitch method and indicate which methods could be used in clinical practice.

## Supplementary Material
