# Supplementary material for: Neural Drive Impairment in Chronic Kidney Disease Patients Is Associated with Neuromuscular Fatigability and Fatigue
Source: Med Sci Sports Exerc. 2022 Dec 13;55(4):727–39. doi: 10.1249/MSS.0000000000003090 (PMC9997639; doi:10.1249/MSS.0000000000003090)
Supplement: Supplementary file 1 [file msse-55-727-s001.pptx]

## Slide 1
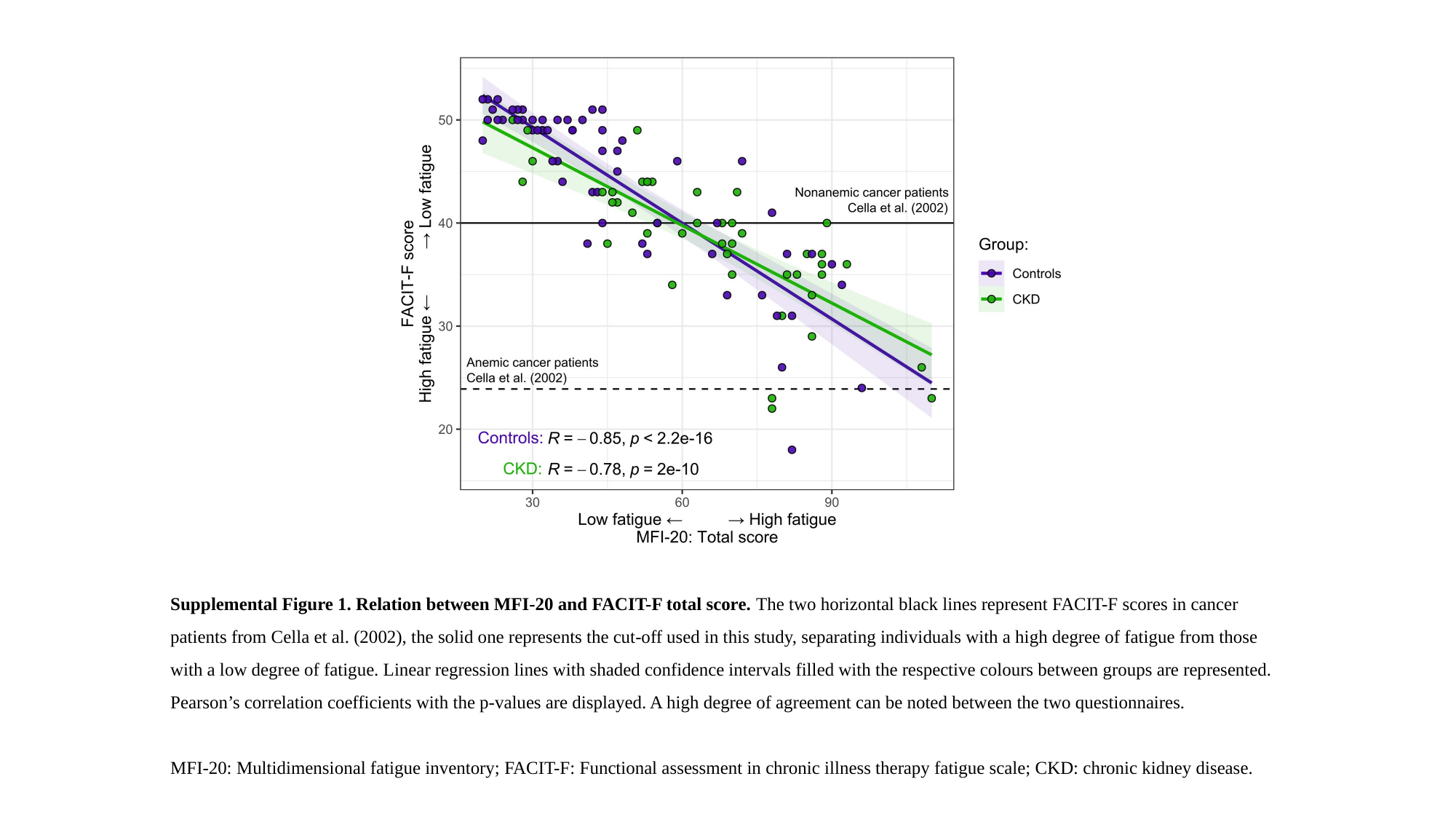

Supplemental Figure 1. Relation between MFI-20 and FACIT-F total score. The two horizontal black lines represent FACIT-F scores in cancer patients from Cella et al. (2002), the solid one represents the cut-off used in this study, separating individuals with a high degree of fatigue from those with a low degree of fatigue. Linear regression lines with shaded confidence intervals filled with the respective colours between groups are represented. Pearson’s correlation coefficients with the p-values are displayed. A high degree of agreement can be noted between the two questionnaires.
MFI-20: Multidimensional fatigue inventory; FACIT-F: Functional assessment in chronic illness therapy fatigue scale; CKD: chronic kidney disease.

## Slide 2
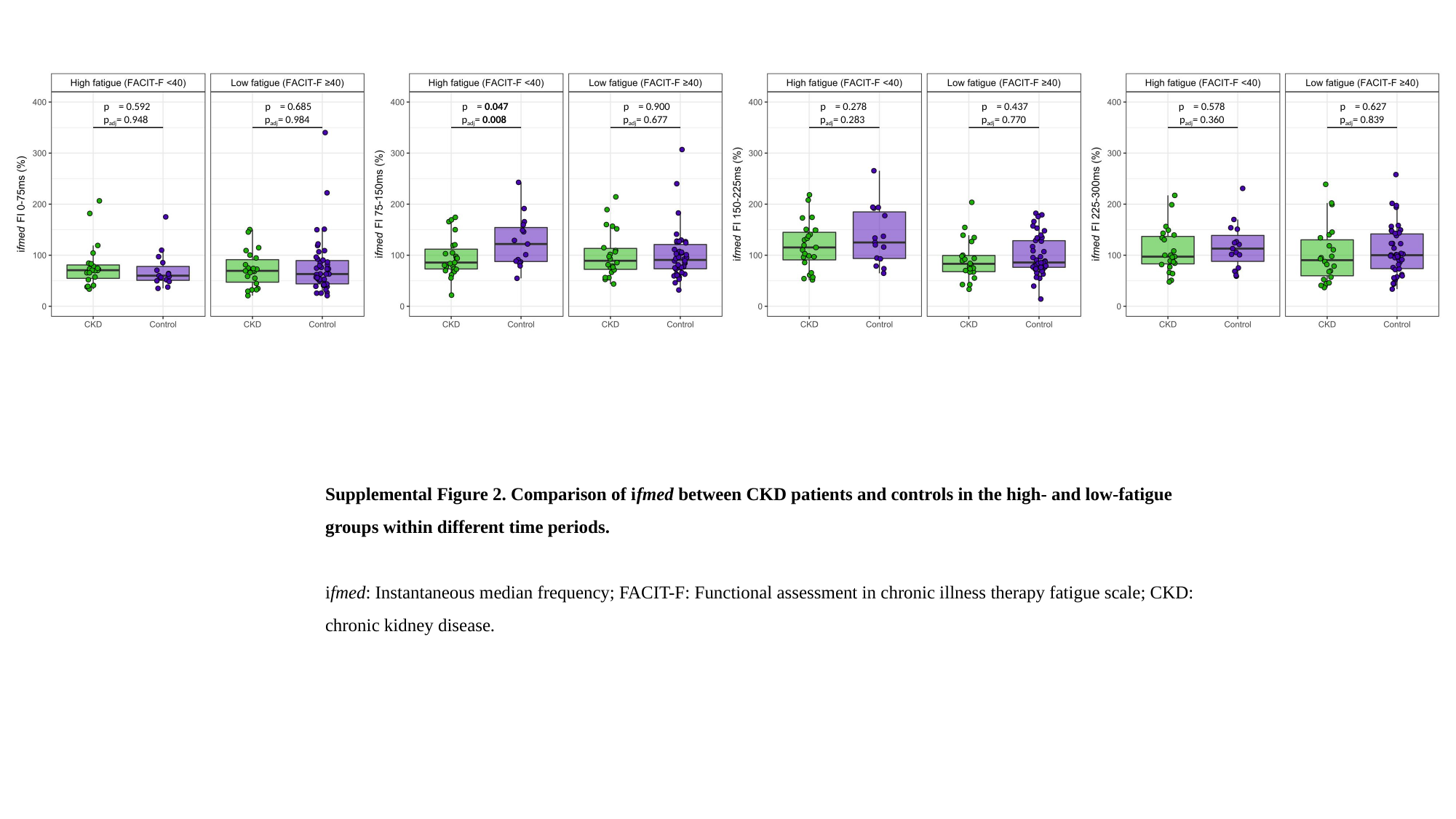

p = 0.047
padj= 0.008
p = 0.900
padj= 0.677
p = 0.278
padj= 0.283
p = 0.437
padj= 0.770
p = 0.578
padj= 0.360
p = 0.627
padj= 0.839
p = 0.592
padj= 0.948
p = 0.685
padj= 0.984
Supplemental Figure 2. Comparison of ifmed between CKD patients and controls in the high- and low-fatigue groups within different time periods.
ifmed: Instantaneous median frequency; FACIT-F: Functional assessment in chronic illness therapy fatigue scale; CKD: chronic kidney disease.

## Slide 3
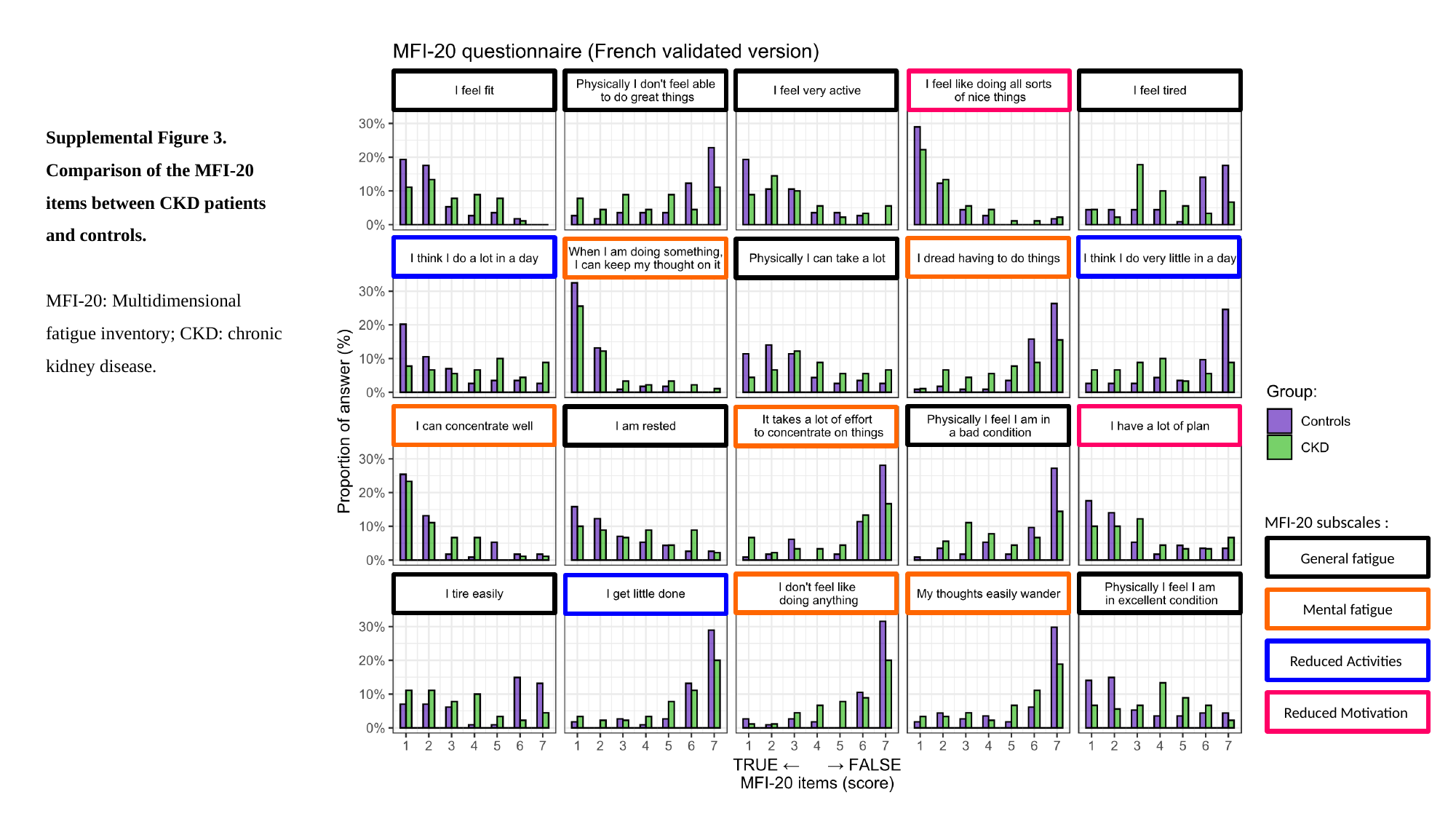

MFI-20 subscales :
General fatigue
Mental fatigue
Reduced Activities
Reduced Motivation
Supplemental Figure 3. Comparison of the MFI-20 items between CKD patients and controls.
MFI-20: Multidimensional fatigue inventory; CKD: chronic kidney disease.

## Slide 4
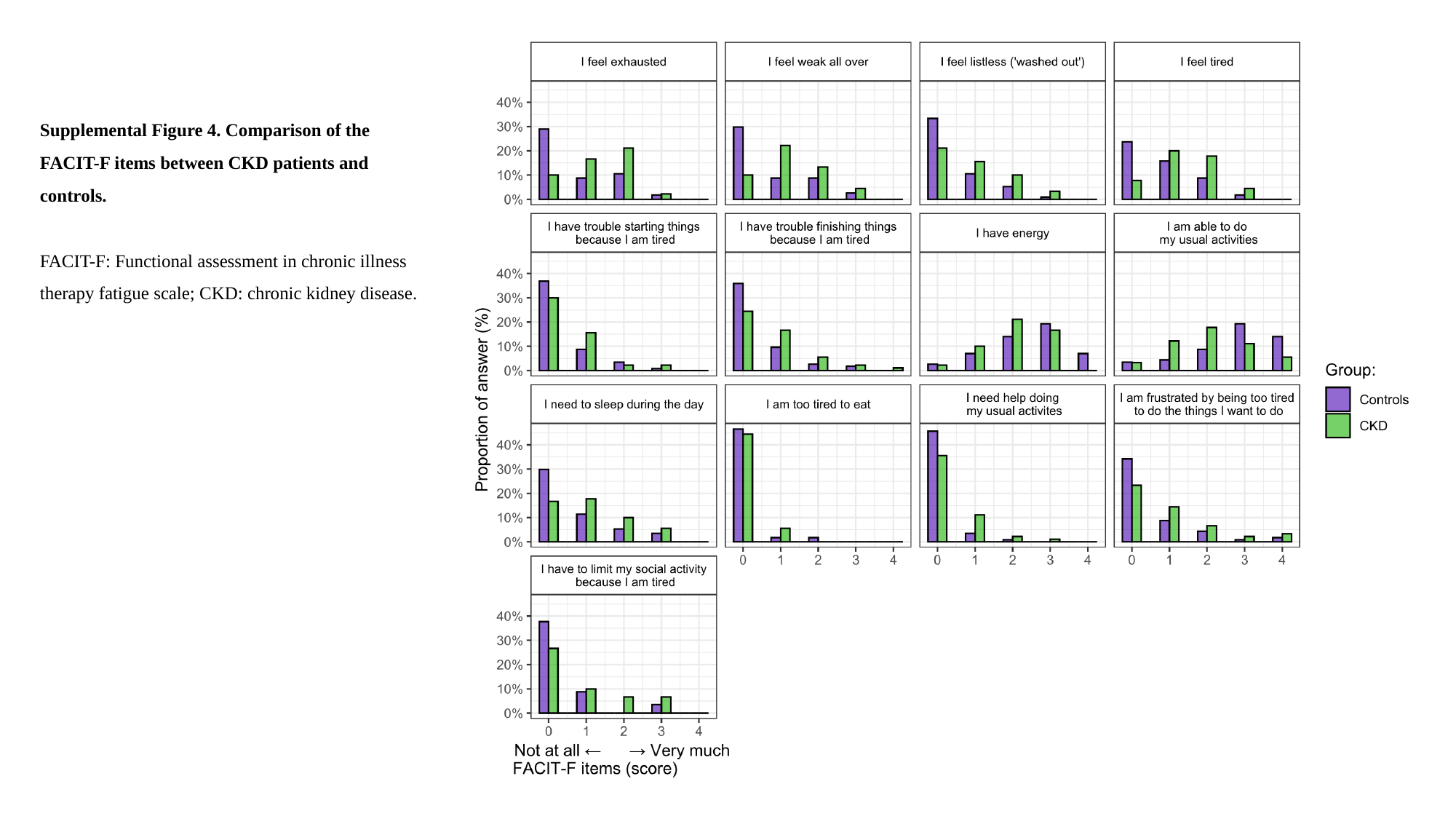

Supplemental Figure 4. Comparison of the FACIT-F items between CKD patients and controls.
FACIT-F: Functional assessment in chronic illness therapy fatigue scale; CKD: chronic kidney disease.
