# Supplementary material for: Neural Drive Impairment in Chronic Kidney Disease Patients Is Associated with Neuromuscular Fatigability and Fatigue
Source: Med Sci Sports Exerc. 2022 Dec 13;55(4):727–39. doi: 10.1249/MSS.0000000000003090 (PMC9997639; doi:10.1249/MSS.0000000000003090)
Supplement: Supplementary file 2 [file msse-55-727-s002.docx]

Supplemental Table 1. Comparison of data between excluded individuals and those included

|  | Excluded | Included | p-value |
| --- | --- | --- | --- |
| n | 59 | 102 |  |
| Age (years), median (IQR) | 75.5 (16.3) | 70.0 (11.0) | **0.024** |
| BMI (kg∙m^2^), median (IQR) | 28.3 (6.1) | 27.8 (5.4) | 0.515 |
| Sex (females), n (%) | 20 (33.9%) | 39 (38.2%) | 0.582 |
| Group  CKD, n (% of group)  Controls, n (% of group) | 26 (36.6%)  33 (36.7%) | 45 (63.4%)  57 (63.3%) | 0.995 |
| eGFR CKD-EPI (ml/min/1.73m^2^), median (IQR) | 21.6 (17.9) | 29.2 (18.3) | 0.549 |
| CKD stages in patients, n (%)  Stage 3B  Stage 4  Stage 5 | 8 (30.8%)  9 (34.6%)  9 (34.6%) | 21 (46.7%)  19 (42.2%)  5 (11.1%) | 0.053 |
| Charlson Comorbidity Index, median (IQR) | 7 (2) | 6 (3) | 0.335 |
| Diabetes, n (%) | 31 (52.5%) | 40 (39.2%) | 0.101 |

IQR: Interquartile range; BMI: Body mass index; CKD: Chronic kidney disease; eGFR CKD-EPI: estimated glomerular filtration rate using the Chronic Kidney Disease EPIdemiology collaboration equation

Supplemental Table 2. Determinants of the neuromuscular estimates during the submaximal contraction at 40 %MVF

|  | **Force integral (%MVF∙s)** |  |  |  | **Mean EMG (%EMG_peak_)** |  |  |  | **i*fmed* (Hz)** |  |  |
| --- | --- | --- | --- | --- | --- | --- | --- | --- | --- | --- | --- |
|  | β | [95%CI] | p-values |  | β | [95%CI] | p-values |  | β | [95%CI] | p-values |
| Contractions, (time) | 0.218 | [0.155, 0.281] | **<0.001** |  | 0.253 | [0.162, 0.345] | **<0.001** |  | -0.244 | [-0.332, -0.156] | **<0.001** |
| Within the block of contraction (1 to 9) $ | 1.943 | [1.638, 2.248] | **<0.001** |  | 0.520 | [0.311, 0.730] | **<0.001** |  | 0.527 | [0.355, 0.698] | **<0.001** |
| Group, (CKD vs. Controls) | -1.866 | [-8.829, 5.097] | 0.599 |  | 2.553 | [-5.952, 11.057] | 0.558 |  | -6.092 | [-17.672, 5.489] | 0.305 |
| Contraction × Group | -0.119 | [-0.215, -0.024] | **0.014** |  | -0.107 | [-0.245, 0.031] | 0.131 |  | -0.026 | [-0.158, 0.106] | 0.698 |
| Block × Group | 0.188 | [-0.271, 0.648] | 0.422 |  | -0.016 | [-0.331, 0.300] | 0.922 |  | -0.135 | [-0.393, 0.123] | 0.309 |
| MFI-20 domains, (score) |  |  |  |  |  |  |  |  |  |  |  |
| General | 0.112 | [-0.137, 0.360] | 0.379 |  | -0.076 | [-0.454, 0.302] | 0.694 |  | -0.367 | [-0.851, 0.116] | 0.140 |
| Mental | -0.115 | [-0.486, 0.257] | 0.545 |  | -0.419 | [-0.983, 0.145] | 0.149 |  | 0.749 | [0.027, 1.471] | **0.045** |
| Motivation | 0.083 | [-0.728, 0.894] | 0.842 |  | 0.125 | [-1.107, 1.357] | 0.843 |  | 0.230 | [-1.347, 1.808] | 0.775 |
| Activity | -0.266 | [-0.808, 0.276] | 0.336 |  | 0.721 | [-0.103, 1.544] | 0.090 |  | 0.052 | [-1.003, 1.106] | 0.924 |

All the model were adjusted for age, sex, body mass index, Charlson comorbidity index and sleep satisfaction.

CKD: chronic kidney disease; BMI: body mass index; MFI: Multidimensional Fatigue Inventory 20 questionnaire; i*fmed*: instantaneous median frequency

$ Each block is composed of 9 submaximal contractions (followed by one explosive and one maximal voluntary contraction), repeated 6 times.

Supplemental Table 3. Determinant of the sEMG parameters during the maximal voluntary contractions of the fatigability protocol

| **Mean EMG (µV)** |  |  |  |  |  |  |  |  |  |  |  |
| --- | --- | --- | --- | --- | --- | --- | --- | --- | --- | --- | --- |
| Models *: | **Overall** |  |  |  | **CKD** |  |  |  | **Controls** |  |  |
|  | β | [95%CI] | p-values |  | β | [95%CI] | p-values |  | β | [95%CI] | p-values |
| Contractions, (time) | -5.153 | [-7.568, -2.738] | **<0.001** |  | -7.920 | [-10.939, -4.902] | **<0.001** |  | -5.153 | [-7.374, -2.932] | **<0.001** |
| Group, (CKD vs. Controls) | -2.564 | [-57.545, 52.417] | 0.927 |  | - |  |  |  | - |  |  |
| Contraction × Group | -2.589 | [-6.225, 1.046] | 0.166 |  | - |  |  |  | - |  |  |
| MFI-20 domains, (score) |  |  |  |  |  |  |  |  |  |  |  |
| General | 0.730 | [-1.591, 3.052] | 0.539 |  | 1.140 | [-2.195, 4.474] | 0.508 |  | 0.155 | [-3.998, 3.689] | 0.938 |
| Mental | 0.223 | [-3.243, 3.689] | 0.900 |  | 0.453 | [-4.429, 5.336] | 0.857 |  | -0.520 | [-6.148, 5.108] | 0.857 |
| Motivation | 2.729 | [-4.843, 10.301] | 0.482 |  | 3.172 | [-7.644, 13.989] | 0.569 |  | 2.506 | [-8.758, 13.770] | 0.665 |
| Activity | -3.478 | [-8.538, 1.582] | 0.181 |  | -3.747 | [-10.895, 3.400] | 0.312 |  | -1.173 | [-8.776, 6.430] | 0.764 |
| **Mean EMG-FI (%)** |  |  |  |  |  |  |  |  |  |  |  |
|  | β | [95%CI] | p-values |  | β | [95%CI] | p-values |  | β | [95%CI] | p-values |
| Contractions, (time) | -2.194 | [-3.824, -0.564] | **0.010** |  | -2.660 | [-4.655, -0.664] | **0.012** |  | -2.204 | [-3.696, -0.712] | **0.005** |
| Group, (CKD vs. Controls) | -11.213 | [-29.110, 6.683] | 0.222 |  | - |  |  |  | - |  |  |
| Contraction × Group | 0.372 | [-2.083, 2.827] | 0.767 |  | - |  |  |  | - |  |  |
| MFI-20 domains, (score) |  |  |  |  |  |  |  |  |  |  |  |
| General | 0.444 | [-0.292, 1.180] | 0.240 |  | 0.536 | [-0.867, 1.939] | 0.460 |  | 0.314 | [-0.803, 1.432] | 0.584 |
| Mental | -0.973 | [-2.073, 0.126] | 0.086 |  | 0.034 | [-2.021, 2.090] | 0.974 |  | -1.662 | [-3.299, -0.024] | 0.052 |
| Motivation | -0.973 | [-3.375, 1.429] | 0.429 |  | -2.618 | [-7.170, 1.933] | 0.268 |  | 0.004 | [-3.272, 3.279] | 0.998 |
| Activity | 1.316 | [-0.290, 2.921] | 0.112 |  | -0.203 | [-3.210, 2.805] | 0.896 |  | 2.799 | [0.587, 5.010] | **0.017** |
| **i*fmed* (Hz)** |  |  |  |  |  |  |  |  |  |  |  |
|  | β | [95%CI] | p-values |  | β | [95%CI] | p-values |  | β | [95%CI] | p-values |
| Contractions, (time) | -1.232 | [-2.063, -0.401] | **0.005** |  | -1.399 | [-2.492, -0.305] | **0.012** |  | -0.901 | [-1.879, 0.768] | 0.071 |
| Group, (CKD vs. Controls) | -4.005 | [-17.192, 9.183] | 0.553 |  | - |  |  |  | - |  |  |
| Contraction × Group | -0.058 | [-1.308, 1.191] | 0.827 |  | - |  |  |  | - |  |  |
| MFI-20 domains, (score) |  |  |  |  |  |  |  |  |  |  |  |
| General | -0.582 | [-1.121, -0.043] | **0.037** |  | 0.066 | [-0.485, 0.617] | 0.813 |  | -0.755 | [-1.267, -0.242] | **0.003** |
| Mental | 1.142 | [0.337, 1.947] | **0.007** |  | 0.566 | [-0.239, 1.371] | 0.168 |  | 0.895 | [0.139, 1.650] | **0.020** |
| Motivation | 0.496 | [-1.263, 2.255] | 0.582 |  | 0.022 | [-1.777, 1.822] | 0.981 |  | 1.437 | [-0.071, 2.945] | 0.062 |
| Activity | -0.004 | [-1.180, 1.171] | 0.995 |  | -1.110 | [-2.298, 0.079] | 0.067 |  | 0.501 | [-0.513, 1.514] | 0.333 |

*** All the model were adjusted for age, sex, BMI, Charlson comorbidity index and sleep satisfaction. CKD and control models were also adjusted for eGFR CKD-EPI no BSA, and CKD models adjusted for haemoglobin level.

CKD: chronic kidney disease; BMI: body mass index; MFI: Multidimensional Fatigue Inventory 20 questionnaire; EMG-FI: fatigability index of the mean EMG; i*fmed*: instantaneous median frequency. A higher MFI-20 score represents greater fatigue.

Supplemental Table 4. Determinant of the absolute RER during the fatigability protocol

| **Raw RER 0-30ms (µV∙s^-1^)** |  |  |  |  |  |  |  |  |  |  |  |
| --- | --- | --- | --- | --- | --- | --- | --- | --- | --- | --- | --- |
| Models *: | **Overall** |  |  |  | **CKD** |  |  |  | **Controls** |  |  |
|  | β | [95%CI] | p-values |  | β | [95%CI] | p-values |  | β | [95%CI] | p-values |
| Contractions, (time) | -71.675 | [-148.828, 5.478] | 0.071 |  | -113.498 | [-200.630, -26.367] | **0.012** |  | -71.675 | [-151.851, 8.502] | 0.085 |
| Group, (CKD vs. Controls) | -363.098 | [-1206.922, 480.727] | 0.401 |  | - |  |  |  | - |  |  |
| Contraction × Group | -46.619 | [-162.777, 69.539] | 0.433 |  | - |  |  |  | - |  |  |
| MFI-20 domains, (score) |  |  |  |  |  |  |  |  |  |  |  |
| General | -5.581 | [-33.939, 22.777] | 0.701 |  | 6.103 | [-33.133, 45.339] | 0.762 |  | -6.872 | [-57.873, 44.130] | 0.793 |
| Mental | -7.582 | [-49.922, 34.757] | 0.726 |  | 27.002 | [-30.450, 84.454] | 0.364 |  | -11.581 | [-86.266, 63.105] | 0.763 |
| Motivation | -28.620 | [-121.125, 63.885] | 0.546 |  | -137.637 | [-264.920, -10.345] | **0.042** |  | 25.811 | [-123.655, 175.278] | 0.737 |
| Activity | -1.605 | [-63.421, 60.211] | 0.960 |  | -10.808 | [-94.915, 73.299] | 0.803 |  | -10.194 | [-111.074, 90.696] | 0.844 |
| **Raw RER 0-50ms (µV∙s^-1^)** |  |  |  |  |  |  |  |  |  |  |  |
|  | β | [95%CI] | p-values |  | β | [95%CI] | p-values |  | β | [95%CI] | p-values |
| Contractions, (time) | -48.448 | [-125.704, 28.809] | 0.219 |  | -121.146 | [-216.048, -26.244] | **0.016** |  | -48.448 | [-121.075, 24.181] | 0.196 |
| Group, (CKD vs. Controls) | -318.511 | [-1197.817, 560.795] | 0.478 |  | - |  |  |  | - |  |  |
| Contraction × Group | -77.044 | [-193.357, 39.269] | 0.195 |  | - |  |  |  | - |  |  |
| MFI-20 domains, (score) |  |  |  |  |  |  |  |  |  |  |  |
| General | -1.791 | [-33.196, 29.614] | 0.911 |  | 8.415 | [-33.900, 50.730] | 0.699 |  | -1.766 | [-59.030, 55.499] | 0.952 |
| Mental | -12.469 | [-59.359, 34.420] | 0.602 |  | 16.070 | [-45.890, 78.030] | 0.615 |  | -15.400 | [-99.257, 55.499] | 0.721 |
| Motivation | -27.950 | [-130.394, 74.495] | 0.593 |  | -151.592 | [-288.862, -14.322] | **0.038** |  | 30.079 | [-137.742, 197.900] | 0.727 |
| Activity | -11.125 | [-79.583, 57.333] | 0.750 |  | -6.928 | [-97.634, 83.778] | 0.882 |  | -26.976 | [-140.255, 86.303] | 0.643 |
| **Raw RER 0-75ms (µV∙s^-1^)** |  |  |  |  |  |  |  |  |  |  |  |
|  | β | [95%CI] | p-values |  | β | [95%CI] | p-values |  | β | [95%CI] | p-values |
| Contractions, (time) | -40.194 | [-99.157, 18.770] | 0.182 |  | -102.375 | [-172.528, -32.222] | **0.006** |  | -40.194 | [-96.368, 15.981] | 0.166 |
| Group, (CKD vs. Controls) | -247.561 | [-933.276, 438.155] | 0.479 |  | - |  |  |  | - |  |  |
| Contraction × Group | -62.181 | [-150.953, 26.591] | 0.170 |  | - |  |  |  | - |  |  |
| MFI-20 domains, (score) |  |  |  |  |  |  |  |  |  |  |  |
| General | 3.927 | [-21.454, 29.308] | 0.762 |  | 10.888 | [-27.094, 48.871] | 0.578 |  | 4.337 | [-39.684, 48.359] | 0.848 |
| Mental | -12.637 | [-50.532, 25.257] | 0.514 |  | 6.615 | [-49.786, 63.015] | 0.820 |  | -20.962 | [-85.426, 43.502] | 0.527 |
| Motivation | -4.150 | [-86.943, 78.643] | 0.922 |  | -109.357 | [-231.653, 12.940] | 0.089 |  | 45.141 | [-83.869, 174.152] | 0.496 |
| Activity | -18.885 | [-74.211, 36.441] | 0.504 |  | -10.931 | [-93.330, 71.468] | 0.796 |  | -28.519 | [-115.601, 58.563] | 0.524 |

*** All the model were adjusted for age, sex, BMI, Charlson comorbidity index and sleep satisfaction. CKD and control models were also adjusted for eGFR CKD-EPI no BSA, and CKD models adjusted for haemoglobin level.

CKD: chronic kidney disease; BMI: body mass index; eGFR CKD-EPI no BSA: estimated glomerular filtration rate using the Chronic Kidney Disease EPIdemiology collaboration equation adjusted for body surface area using the Haycock Method (Haycock et al., 1978); MFI: Multidimensional Fatigue Inventory 20 questionnaire; RER: rate of electromyography rise calculated on raw signal. A higher MFI-20 score represents greater fatigue.

Supplemental Table 5. Determinant of the i*fmed* during explosive contractions considering all the different time period during the fatigability protocol

| **i*fmed* (Hz)** |  |  |  |  |  |  |  |  |  |  |  |
| --- | --- | --- | --- | --- | --- | --- | --- | --- | --- | --- | --- |
| Models *: | **Overall** |  |  |  | **CKD** |  |  |  | **Controls** |  |  |
|  | β | [95%CI] | p-values |  | β | [95%CI] | p-values |  | β | [95%CI] | p-values |
| Contractions, (time) | -1.948 | [-2.895, -1.002] | **<0.001** |  | -1.861 | [-2.940, -0.782] | **0.002** |  | -1.948 | [-2.889, -1.008] | **<0.001** |
| Time-window (0-75 to 225-300ms) | 7.804 | [6.179, 9.430] | **<0.001** |  | 8.238 | [7.186, 9.291] | **<0.001** |  | 7.804 | [6.867, 8.742] | **<0.001** |
| Group, (CKD vs. Controls) | -4.485 | [-15.057, 6.086] | 0.407 |  | - |  |  |  |  |  |  |
| Contraction × Group | -0.017 | [-1.443, 1.408] | 0.981 |  | - |  |  |  |  |  |  |
| Time-window × Group | 0.429 | [-2.018, 2.877] | 0.732 |  | - |  |  |  |  |  |  |
| MFI-20 domains, (score) |  |  |  |  |  |  |  |  |  |  |  |
| General | -0.190 | [-0.585, 0.206] | 0.350 |  | 0.120 | [-0.682, 0.923] | 0.771 |  | -0.324 | [-1.042, 0.394] | 0.381 |
| Mental | 0.500 | [-0.091, 1.090] | 0.101 |  | 0.366 | [-0.768, 1.501] | 0.531 |  | 0.596 | [-0.452, 1.643] | 0.271 |
| Motivation | 0.057 | [-1.234, 1.348] | 0.931 |  | 0.883 | [-1.704, 3.471] | 0.508 |  | 0.093 | [-1.967, 2.153] | 0.930 |
| Activity | 0.038 | [-0.825, 0.901] | 0.931 |  | -0.496 | [-2.174, 1.181] | 0.566 |  | 0.458 | [-0.983, 1.900] | 0.536 |

*** All the model were adjusted for age, sex, BMI, Charlson comorbidity index and sleep satisfaction. CKD and control models were also adjusted for eGFR CKD-EPI no BSA, and CKD models adjusted for haemoglobin level.

CKD: chronic kidney disease; BMI: body mass index; eGFR CKD-EPI no BSA: estimated glomerular filtration rate using the Chronic Kidney Disease EPIdemiology collaboration equation adjusted for body surface area using the Haycock Method (Haycock et al., 1978); MFI: Multidimensional Fatigue Inventory 20 questionnaire; i*fmed*: instantaneous median frequency defined as the pseudo-frequency that divides the EMG signal spectrum into two parts with equal energy. The i*fmed* was then averaged within the following time-period: 0-75, 75-150, 150-225 and 225-300ms.
